# Statistical Optimization for Acid Hydrolysis of Microcrystalline Cellulose and Its Physiochemical Characterization by Using Metal Ion Catalyst

**DOI:** 10.3390/ma7106982

**Published:** 2014-10-13

**Authors:** Md. Ziaul Karim, Zaira Zaman Chowdhury, Sharifah Bee Abd Hamid, Md. Eaqub Ali

**Affiliations:** Nanotechnology and Catalysis Center (NANOCAT), University Malaya, Kuala Lumpur 50603, Malaysia; E-Mails: ziaul@um.edu.my (M.Z.K.); zaira.chowdhury76@gmail.com (Z.Z.C.); eaqubali@um.edu.my (M.E.A.)

**Keywords:** microcrystalline cellulose, hydrocellulose, response surface methodology, percentage of crystallinity, amorphous sections

## Abstract

Hydrolyzing the amorphous region while keeping the crystalline region unaltered is the key technology for producing nanocellulose. This study investigated if the dissolution properties of the amorphous region of microcrystalline cellulose can be enhanced in the presence of Fe^3+^ salt in acidic medium. The process parameters, including temperature, time and the concentration of metal chloride catalyst (FeCl_3_), were optimized by using the response surface methodology (RSM). The experimental observation demonstrated that temperature and time play vital roles in hydrolyzing the amorphous sections of cellulose. This would yield hydrocellulose with higher crystallinity. The factors that were varied for the production of hydrocellulose were the temperature (*x*_1_), time (*x*_2_) and FeCl_3_ catalyst concentration (*x*_3_). Responses were measured in terms of percentage of crystallinity (*y*_1_) and the yield (*y*_2_) of the prepared hydrocellulose. Relevant mathematical models were developed. Analysis of variance (ANOVA) was carried out to obtain the most significant factors influencing the responses of the percentage of crystallinity and yield. Under optimum conditions, the percentage of crystallinity and yield were 83.46% and 86.98% respectively, at 90.95 °C, 6 h, with a catalyst concentration of 1 M. The physiochemical characteristics of the prepared hydrocellulose were determined in terms of XRD, SEM, TGA and FTIR analyses. The addition of FeCl_3_ salt in acid hydrolyzing medium is a novel technique for substantially increasing crystallinity with a significant morphological change.

## 1. Introduction

Microcrystalline cellulose (MCC) is a porous, white, odorless, crystalline powder, which has been derived by partial depolymerization of cellulose [1]. It is obtained by the hydrolysis of wood or cotton linters using dilute mineral acid. In this context, the pulp is initially immersed in hot dilute acid, which dissolves the amorphous segments of the cellulosic chain, leaving the micro fibrils exhibiting a microcrystalline texture [2]. After achieving a certain degree of polymerization, the samples are withdrawn from the acid hydrolysis bath, washed, dried and ground to yield microcrystalline cellulose with a specific particle size and moisture content. It is chemically inactive and hydroscopic, having a particle size of usually 20–80 µm, with a degree of polymerization lower than 350 [3,4]. Due to its insolubilities in common reagents, like water, organic solvent and dilute acids, its lubricating properties, as well as its hygroscopic tendencies, it has been used in the cosmetics and food industries as a fat replacement [5,6]. It is used extensively by pharmaceutical industries as diluents or binders for oral tablets [7]. Furthermore, it is used for the dry and wet formulation of capsules. It is also used for pelletization during the direct compression process [7].

The major sources of MCC are wood pulp and cotton fiber. Recently, some other non-woody biomass, such as soybean, corn stalk, oath and rice hulls, as well as sugar beet pulp [8,9], bagasse and maize cob [10,11], wheat, barley and oath straw [12], groundnut shell and rice husks [13], reed stalks [14] and cereal straw [15], have been used. Indian bamboo [16] and *Luffa cylindrica* [17] have been identified as prospective sources of MCC. During the acid hydrolysis of cellulosic materials, amorphous regions are disintegrated, resulting in a highly crystalline substrate with different degrees of the crystallinity index [18]. The amorphous regions are selectively hydrolyzed by strong mineral acids, such as hydrochloric, nitric and sulfuric acids [19,20]. However, to ensure the degradation of amorphous regions while keeping the crystalline region intact can enhance the yield and crystallinity of the MCC. Previous researchers used different types of transition metal salt catalyst, such as FeCl_3_, CuCl_2_ and AlCl_3_, during acid hydrolysis of cellulose [21]. Another finding showed that a minute amount of FeSO_4_ can enhance the reducing sugar content during the hydrolysis of coniferous sawdust [22]. It has been reported that hydrolysis efficiency can be improved significantly by the presence of Fe^3+^ and H^+^ ions simultaneously in the reaction medium [23]. Several studies have been conducted for the extraction of cellulose from different types of biomass. However, limited studies were performed to pretreat microcrystalline cellulose to improve its crystallinity by the transition metal ion catalyzed acid hydrolysis process by optimizing the process parameters by statistical regression analysis.

RSM is a combination of mathematical and statistical analyses of experimental results that can establish an empirical relationship between process variables with desired responses or product characteristics. It provides a complete experimental design for data exploration, model fitting, as well as process optimization [24,25]. The main goal of this study is to pretreat the MCC in the presence of FeCl_3_ catalyst during the acid hydrolysis process. Three individual factors of temperature, time and concentration of metal ion catalyst were selected to investigate the hydrolysis mechanism of MCC in hydrochloric acid medium. The responses measured were the crystallinity of the hydrocellulose and the yield, to develop corresponding mathematical models. The effect of the process variables on both of the responses was analyzed in terms of ANOVA analysis. The upcoming perspective of this research is to apply the pretreated MCC to lab-scale, as well as large-scale pilot plant experiments to produce nanocellulose with the desired properties.

## 2. Results and Discussion

### 2.1. Development of Regression Model

Two regression models depicting the crystallinity index and the yield for the prepared hydrocellulose were developed. The models were carefully selected based on the highest order polynomials. The additional terms were significant, as well as the models were not aliased based on the sequential model sum of squares [26]. Model fitting parameters describing the reaction conditions were calculated from Table 1 illustrating the design matrix in terms of actual and coded factors. For the crystallinity index and yield, a quadratic model was developed. Equations (1) and (2) represent the final empirical equations expressed by using the coded factor.

(1) $$y_{1}=83.12+4.45x_{1}+2.35x_{2}+0.62x_{3}+0.60x_{1}x_{2}+0.096x_{2}x_{3}+0.29x_{3}x_{1}-1.42x_{1}^{2}+0.97x_{2}^{2}+0.58x_{3}^{2}$$

(2) $$y_{2}=82.36-5.29x_{1}-2.07x_{2}-1.71x_{3}-0.92x_{1}x_{2}+0.11x_{2}x_{3}-0.25x_{1}x_{3}+1.04x_{1}^{2}+0.56x_{2}^{2}+1.38x_{3}^{2}$$

The coefficient with linear terms of temperature (*x*_1_), time (*x*_2_) and catalyst concentration (*x*_3_) shows the effect of that particular factor for the acid hydrolysis of MCC. On the contrary, the coefficient multiplied by two factors, such as *x*_1_*x*_2_, *x*_2_*x*_3_ and *x*_3_*x*_1_, depicts the interaction effects on the responses. Second order terms related to $x_{1}^{2}$, $x_{2}^{2}$ and $x_{3}^{2}$ represent the quadratic effect. A positive sign indicates a synergistic effect, whereas a negative sign indicates an antagonistic effect.

**Figure 1:**
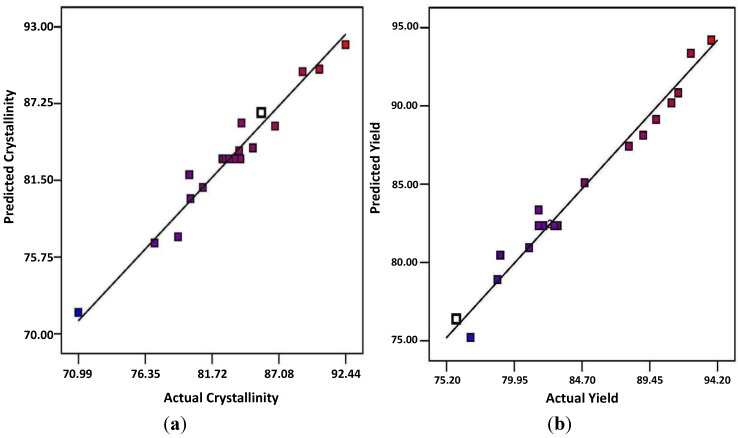
Predicted *versus* actual: (**a**) percentage of crystallinity; (**b**) yield of acid hydrolyzed microcrystalline cellulose (MCC).

Figure 1a,b shows the linear plots of predicted *versus* experimental percentage of crystallinity and yield, respectively. As can be observed from these two plots, the predicted values for the crystallinity index and MCC yield were closer to their experimental values. This was due to their large *R*^2^ values, which were almost near unity. The *R*^2^ value for Equations (1) and (2) were 0.96 and 0.97 for the percentage of crystallinity (Figure 1a) and yield (Figure 1b), respectively. This certifies the excellent adjustment of the developed models with the experimental data.

### 2.2. ANOVA Analysis and Lack of Fit

The experimental results obtained were examined by analysis of variance (ANOVA). This statistical test aids in measuring the accuracy of the developed model. The regression coefficients of the polynomial response surface models, corresponding *R*^2^ values and lack of fit tests are provided in Table 1.

**Table 1:** Statistical parameters for ANOVA for the model regression for the percentage of crystallinity and yield.

Statistical parameters	Percentage of crystallinity *y*_1_	Yield *y*_2_
Standard Deviation, SD%	1.29	1.18
Correlation Coefficient, *R*^2^	0.96	0.97
Adjusted *R*^2^	0.93	0.94
Mean	83.20	84.39
Coefficient of variation, CV	1.55	1.40
Adequate precision	22.04	22.74

The quality of the models can be further verified by observing the correlation coefficient *R*^2^ and standard deviation. Table 1 shows that the experimental *R*^2^ was quite close to the adjusted *R*^2^. Moreover, the small values of the coefficient of variation (CV), as well as the standard deviation reflect the reproducibility of the model. The signal-to-noise ratio is determined in terms of adequate precision. For successful resolution of the model, this value should be greater than four. The adequate precision obtained for crystallinity and yield were 22.04 and 22.74. This showed that the developed models could be used to navigate the design.

The significance of linear, interaction and quadratic model terms was determined using the *F*-test and *p*-value, as presented in Table 2 and Table 3. The most significant variable influencing the percentage of crystallinity and yield was the linear term of temperature. The results demonstrated that the regression models for the responses of yield and crystallinity percentage were significant by the *F*-test at the 5% confidence level. Analysis of variance also confirmed that the models were highly significant, as the probability (*p*) values of all regression models were less than 0.003. The *R*^2^ values for these response variables were higher than 0.80 (0.922–0.975), which ensures a satisfactory fitness of the regression models with the experimental data.

**Table 2:** ANOVA analysis and lack of fit test for the response surface model for the percentage of crystallinity (*y*_1_).

Source	Sum of squares	Degrees of freedom	Mean square	*F*-Value	Prob > *F*	Comments
Model	408.31	9	45.37	27.39	<0.0001	Significant
*x*_1_	270.85	1	270.85	163.53	<0.0001	–
*x*_2_	75.66	1	75.66	45.68	<0.0001	–
*x*_3_	5.25	1	5.25	3.17	0.1054	–
$x_{1}^{2}$	29.22	1	29.22	17.64	0.0018	–
$x_{2}^{2}$	13.70	1	13.70	8.27	0.0165	–
$x_{3}^{3}$	4.80	1	4.80	2.90	0.1195	–
*x*_1_*x*_2_	2.84	1	2.84	1.72	0.2194	–
*x*_1_*x*_3_	0.68	1	0.68	0.41	0.5365	–
*x*_2_*x*_3_	0.074	1	0.074	0.045	0.8367	–
Residuals	16.56	10	1.66	–	–	–
Lack of fit	15.18	5	3.04	11.01	0.0099	Significant
Pure error	1.38	5	0.28	–	–	–

**Table 3:** ANOVA analysis and lack of fit test for the response surface model for the percentage yield (*y*_2_).

Source	Sum of squares	Degrees of freedom	Mean square	*F*-Value	Prob > *F*	Comments
Model	529.71	9	58.86	42.18	<0.0001	Significant
*x*_1_	382.84	1	382.84	274.38	<0.0001	–
*x*_2_	58.45	1	58.45	41.89	<0.0001	–
*x*_3_	40.17	1	40.17	28.79	0.0003	–
$x_{1}^{2}$	15.60	1	15.60	11.18	0.0074	–
$x_{2}^{2}$	4.57	1	4.57	3.28	0.1004	–
$x_{3}^{3}$	27.30	1	27.30	19.57	0.0013	–
*x*_1_*x*_2_	1.40	1	1.40	4.83	0.0527	–
*x*_1_*x*_3_	2.43	1	2.43	0.35	0.5666	–
*x*_2_*x*_3_	0.36	1	0.36	0.073	0.7931	–
Residuals	13.95	10	1.40	–	–	–
Lack of fit	12.15	5	2.43	6.74	<0.0282	Significant
Pure error	1.80	5	0.36	–	–	–

The value of probable *F* is less than 0.0001 for percentage of crystallinity (*y*_1_), reflecting the significance of the model. From Table 2, it is observed that temperature (*x*_1_), pretreatment time (*x*_2_) and their quadratic terms of ($x_{1}^{2}$) and ($x_{2}^{2}$) are significant model terms. Referring to Table 3 for the quadratic model of MCC yield, *x*_1_, *x*_2_, *x*_3_, as well as quadratic terms of $x_{1}^{2}\;$ and $x_{3}^{2}$ are significant model terms, whereas other interaction terms are negligible relative to the response. For the percentage of crystallinity (*y*_1_) yield (*y*_2_), temperature was found to have the greatest effect on these responses by showing the highest *F*-value of 270.85 and 382.84, respectively, as shown in Table 2 and Table 3. Hydrolyzing time was significant to the responses, but not as noteworthy compared to temperature. Catalyst concentration had a moderate effect, whereas the interaction effect of temperature and time (*x*_1_*x*_2_ = 2.84) and temperature and catalyst concentration (*x*_1_*x*_3_ = 2.43) were more pronounced than the other two interaction terms relative to the percentage of crystallinity and yield, respectively.

## 2.3. Process Variables Optimization

A numerical optimization is also carried out for both responses. Overall optimal conditions are evaluated. The criteria applied for graphical optimization are to maximize the yield and crystallinity index of the product by keeping the reaction variables within the range studied. Under the optimum condition, the corresponding predicted response values of the percentage of crystallinity and yield are 82.37 and 88.41, respectively. The sample has been prepared under optimum conditions, and the experimental results are compared with the predicted values. The percentage of deviation between the predicted and experimental conditions is evaluated and presented in Table 4.

**Table 4:** Process parameter optimization for the acid hydrolysis of MCC in the presence of FeCl_3_.

Hydrolysis temperature (°C)	Hydrolysis time (h)	Catalyst concentration (M)	Percentage of crystallinity (*y*_1_)			Percentage yield (*y*_2_)		
			Predicted	Experimental	Error	Predicted	Experimental	Error
90.99	6	1	82.37	83.46	1.32	88.41	86.98	1.62

Under the same experimental conditions of temperature and time (Table 4), the experiment was conducted without FeCl_3_ catalyst in the presence of HCl acid (2.5 M) only. The percentage of crystallinity obtained was 79.25%. However, in the presence of both catalyst and acid together, the percentage of crystallinity increased significantly up to 83.46%. A similar experiment was also conducted using optimum conditions of temperature and time (Table 4), in the presence of H_2_SO_4_ (2.5 M) and Fe_2_(SO_4_)_3_ (1 M). However, the percentage of crystallinity obtained was 51.84%. The lower crystallinity obtained from sulfuric acid can be explained due to it being a weaker acid with lower K_a_ value, thus dissociating in water sparingly. Sulfate is a stronger conjugate base than chloride in water.

**Figure 2:**
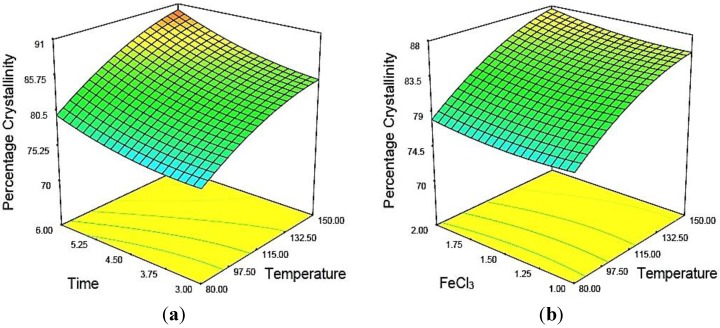
Response surface and contour plots of the combined effects of: (**a**) temperature and time; (**b**) temperature and catalyst concentration on the percentage of crystallinity (*y*_1_) of acid hydrolyzed MCC when the other two variables were at center points.

Figure 2a was constructed to show the effects of temperature and time on the percentage of crystallinity of MCC, where catalyst concentration was kept constant at 1.5. Figure 2b depicts the three-dimensional response surfaces with a contour plot that shows the combined effects of two significant variables, hydrolysis temperature and time on the percentage of crystallinity, where the hydrolyzing time was fixed at the zero level, which was 4.5 h. In this work, all three variables studied were found to have synergistic effects on the percentage of crystallinity of MCC. The percentage of crystallinity was increased significantly when the temperature and time were maximal (Figure 2a). This was expected, as the progressive increase of temperature and hydrolyzing time would increase the diffusion of acids into the amorphous region of cellulose. Enhanced contact time would cause physical swelling of MCC. Consequently, the surface area of the sample would increase, resulting in higher hydrolysis efficiency. Increasing the concentration of the metal ion catalyst increased the percentage of crystallinity. Basically, Fe^3+^ can form a coordination complex with water to yield H^+^ ions, according to Equation (3):

*x* Fe^3+^ + *y*H_2_O → Fe*_x_* (OH)*_y_*^(3*x* − *y*)+^ + *y*H^+^ (3)

H^+^ ions will increase the acidity of the solution, which would disintegrate the glycosidic linkage between glucose units of cellulose. The oxygen atoms of the glucose unit in cellulose can readily form an intermediate complex by absorbing Fe^3+^ ions. This would increase the pyranose bond length and bond angle. Thus, the activation energy would be lowered, resulting in greater hydrolysis efficiency [27,28].

**Figure 3:**
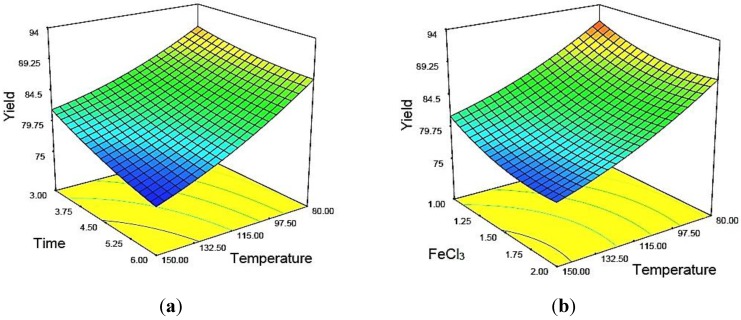
Response surface and contour plots of the combined effects of: (**a**) temperature and time; (**b**) temperature and catalyst concentration on the percentage yield (*y*_2_) of acid hydrolyzed MCC when the other two variables were at center points.

Figure 3a,b shows the three-dimensional response surfaces that were constructed to reveal the effects of catalytic acid hydrolysis reaction variables on MCC yield. Figure 3a represents the combined effect of temperature and time on the response, where the catalyst concentration was fixed at the zero level (1.5). Figure 3b illustrates the effect of temperature and catalyst concentration on the same response, where time was fixed at the zero leve1 (4.5 h). In general, yield was found to decrease with increasing temperature, time and catalyst concentration. As can be seen from both plots (Figure 3a,b), temperature was more dominant relative to the yield as compared to the other two variables. The lowest yield was obtained when the temperature was at the maximum point (173.86 °C, Sample 10) within the studied range, as depicted by the design matrix of Table 5. This was due to the chemical degradation of cellulose in acid hydrolyzing medium [27,28].

## 2.4. Physiochemical Characterization of the Hydrocellulose

**Figure 4:**
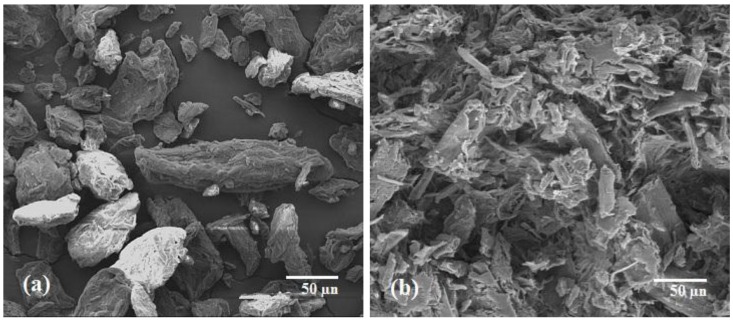
SEM images: (**a**) untreated MCC; and (**b**) acid hydrolyzed MCC in the presence of FeCl_3_ catalyst.

The changes in the structures of the treated MCC particles due to acid hydrolysis in the presence of FeCl_3_ catalyst were confirmed by the images obtained from scanning electron microscopy (Figure 4).

Figure 4a shows the structure of untreated MCC, which was comparatively irregular, flat and rod-like aggregates. The untreated MCC contained a rough surface with numerous individual cellulose whiskers connected by strong hydrogen bonding [29,30,31]. Figure 4b shows images of acid-treated MCC, which were swollen, resulting in the MCC being partially digested by the acid. The aggregated MCC particles were fragmented due to chemical swelling and acid hydrolysis. Minute amounts of holes were observed over the surface of the treated MCC, resulting in a higher hydrolysis rate.

**Figure 5:**
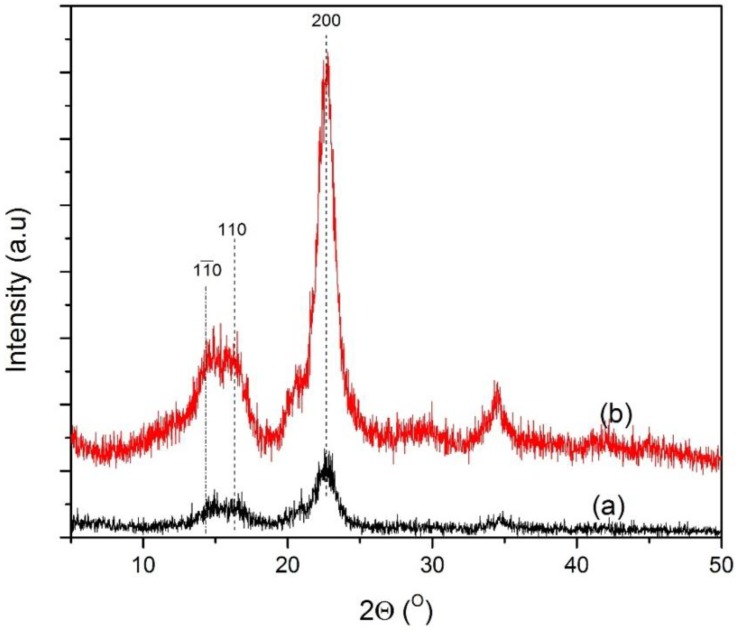
Microcrystalline cellulose: (**a**) untreated MCC; and (**b**) acid hydrolyzed in the presence of FeCl_3_ catalyst. The percentage of crystallinity of the untreated MCC was determined as 56.05%, and this value increased to 83.46% after treatment with FeCl_3_.

The XRD patterns of the untreated MCC and pretreated MCC are illustrated in Figure 5. It was reported earlier that the major diffraction peak for cellulose can be observed for 2θ ranging between 22° and 23° as the primary peak, whereas a secondary peak is in the range of 16° to 18° [32,33]. From the XRD distribution pattern, noticeable peaks can be observed within the mentioned range for both the untreated and pretreated MCC. This reflects the presence of the crystalline and amorphous structure of the cellulose constituent. Similar types of curves and peaks were observed for both samples. It seems that catalytic acid hydrolysis did not disrupt the whole structure of the cellulosic matrix. However, the pretreated sample showed higher peak intensity, which might be due to the partial breaking up of glycoside linkages inside the amorphous region, whereas the crystalline region was almost unaltered. The percentage of crystallinity observed for untreated and treated samples under optimum conditions were 56.05% and 83.46%, respectively.

**Table 5:** Experimental design matrix for acid hydrolysis of microcrystalline cellulose (MCC) in the presence of FeCl_3_ catalyst.

Sample ID	Run	Type of point	Level (coded factors)			Reaction variables (actual factors)			Percentage of crystallinity	Percentage yield
						Temperature *x*_1_, (°C)	Time, *x*_2_, (h)	Catalyst concentration, *x*_3_ (M)	*y*_1_	*y*_2_
S-1	1	Fact	−1	−1	−1	150	6	2	92.44	76.89
S-2	2	Fact	+1	−1	−1	150	6	1	88.99	78.77
S-3	3	Fact	−1	+1	−1	80	6	2	80.99	88.99
S-4	4	Fact	+1	+1	−1	80	3	2	78.99	90.99
S-5	5	Fact	−1	−1	+1	80	3	1	77.09	92.33
S-6	6	Fact	+1	−1	+1	150	3	2	86.77	80.99
S-7	7	Fact	−1	+1	+1	80	6	1	79.99	91.45
S-8	8	Fact	+1	+1	+1	150	3	1	84.99	84.89
S-9	9	Axial	−1.412	0	0	56.14	4.5	1.5	70.99	93.78
S-10	10	Axial	+1.412	0	0	173.86	4.5	1.5	85.67	75.89
S-11	11	Axial	0	−1.412	0	115	1.98	4.5	79.89	87.99
S-12	12	Axial	0	+1.412	0	115	7.02	4.5	90.34	78.98
S-13	13	Axial	0	0	−1.412	115	4.5	0.66	83.89	83.89
S-14	14	Axial	0	0	+1.412	115	4.5	2.34	84.09	84.09
S-15	15	Center	0	0	0	115	4.5	1.5	83.99	81.88
S-16	16	Center	0	0	0	115	4.5	1.5	83.09	82.77
S-17	17	Center	0	0	0	115	4.5	1.5	82.78	81.67
S-18	18	Center	0	0	0	115	4.5	1.5	82.99	82.99
S-19	19	Center	0	0	0	115	4.5	1.5	82.58	81.99
S-20	20	Center	0	0	0	115	4.5	1.5	83.55	82.99

**Figure 6:**
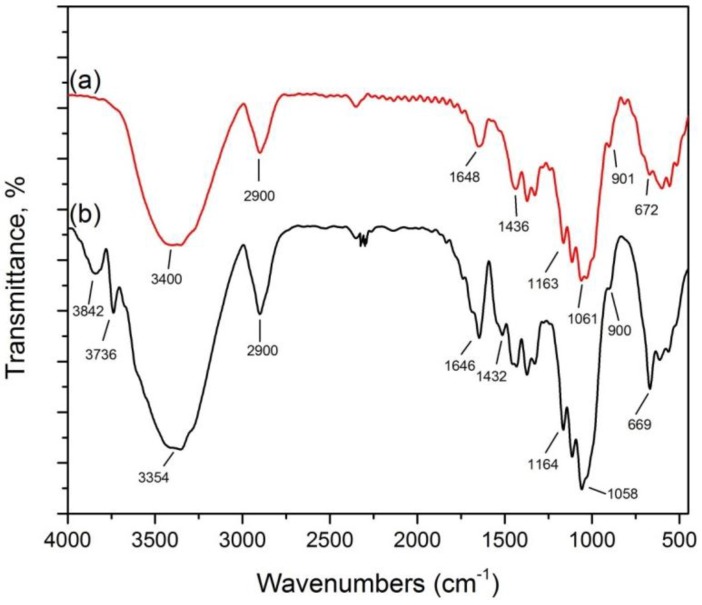
Microcrystalline cellulose: (**a**) untreated MCC; and (**b**) acid hydrolyzed in the presence of FeCl_3_ catalyst.

The FTIR spectra of the MCC samples (Figure 6a) and pretreated MCC sample (Figure 6b) are shown in Figure 6. The FTIR spectra obtained before and after acid swelling in the presence of catalyst revealed the absence of strong chemical reactions. This showed that the treatment could not change the chemical structure of the cellulosic fragments [32]. The peaks around 3400, 2900, 1400 and 900 cm^−1^ in the untreated and treated samples exhibited native cellulose I [32]. The peaks around 672 cm^−1^ changed their frequency level and were observed around 669 cm^−1^ in the pretreated sample. The peaks around 901 and 900 cm^−1^ showed the rocking vibration of the –C–H band in cellulose. The band at 1163 and 1164 cm^−1^ ascribed to the –C–O–C– stretch of the β-1, 4-glycosidic linkage is prominent for both untreated and pretreated MCC samples. The bands at 1436 and 1432 cm^−1^ endorsed the asymmetric bending and wagging of the –CH_2_ group. This showed the intermolecular hydrogen attraction at the C_6_ group [34]. Due to the absorption of water, a small sharp peak around 1648 and 1646 cm^−1^ was identified in both samples [35].

The peak at 2900 cm^−1^ represented the C–H stretching in both samples. The broad absorption peak in the range of 3000 to 3600 cm^−1^ in both samples represented the stretching of the H-bonded –OH groups. Two additional minor peaks around 3736 cm^−1^ and 3842 cm^−1^ appeared for the –OH groups in pretreated sample.

**Figure 7:**
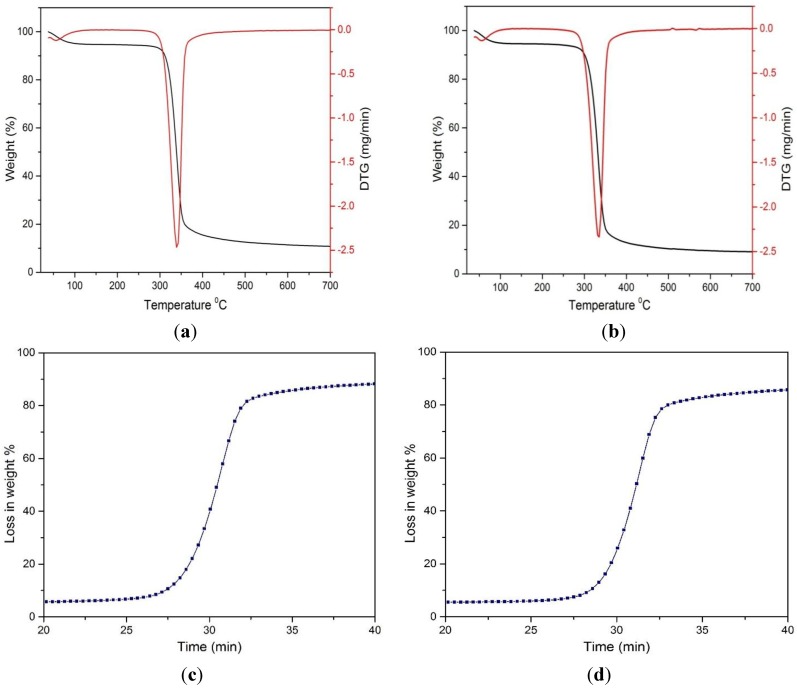
Thermogravimetric analysis of: (**a**) untreated MCC; (**b**) hydrolyzed MCC; and relation between weight loss % and time of (**c**) untreated MCC; (**d**) treated MCC in the presence of FeCl_3_ catalyst.

Thermogravimetric (TGA) and derivative thermogram (DTA) analyses were performed for untreated MCC and the FeCl_3_-catalyzed acid hydrolyzed MCC sample. The TGA and DTA curves obtained are illustrated in Figure 7.

The thermal degradation data (onset temperature, T_on_, 10% weight loss temperature, T_10%_, and 50% weight loss temperature, T_50%_), along with the residual weight loss around 700 °C, including the peak degradation temperature, are provided in Table 6.

**Table 6:** Thermogravimetric analysis of untreated and treated MCC.

Sample	Degradation temperature °C			DTG peak temperature	Residual weight loss at 700 °C
	T_on_	T_10%_	T_50%_	T_max_	
MCC	258	310	333	332	9.00
Treated MCC	280	320	341	339	10.58

It is evident from the curves that initially, the loss of weight occurred in the range of 100–120 °C, which is associated with the moisture content of the sample. After that, the loss in weight of the cellulosic materials increased to a greater extent. However, the loss in weight percentages was greater for untreated sample than the treated one. This is obvious for the treated sample: the disordered amorphous region was decreased with the increase of the hydrogen bond crystalline region of the cellulosic matrix. Referring to the table, it was observed that chemical swelling and acid hydrolysis increased the onset temperature, T_on_, 10% weight loss temperature, T_10%_, and 50% weight loss temperature, T_50%_. This was supported earlier by XRD data, where it was observed that for the treated MCC sample, the crystallinity index was increased, resulting in a higher onset degradation temperature.

**Figure 8:**
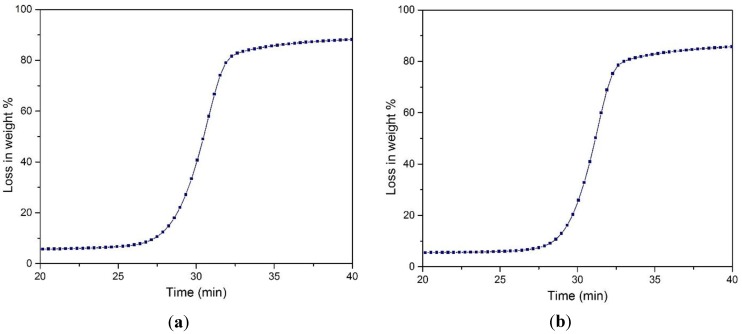
TGA analysis (**a**) the relation between ln(W_0_ − W_inf._)/(W_t_ − W_inf._) and time for the untreated MCC (**b**) the relation between ln(W_0_ − W_inf._)/(W_t_ − W_inf._) and time for the treated MCC.

The data obtained from TGA analysis were used to calculate the activation energy. The value ln(W_0_ − W_inf._/W_t_ − W_inf._) was plotted against time (t) and is shown in Figure 8. Here:

W_0_ = Initial weight of the sample MCC
W_t_ = Weight loss at time t
W_inf._ = Weight of ash

From the figures, it was observed that there are mainly two parts; the first one is very quick, reflecting moisture loss within the initial 30 min, whereas the second one is due to thermal decomposition. The linearization of the plot gave a slope and intercepts. The slope rate constant was calculated to give the activation energy [36,37]. The activation energy calculated for untreated MCC was 185.66 kJ/mole, whereas for the treated one, it was around 211.38 kJ/mole. This can be ascribed to the degradation of the amorphous region of cellulose, resulting in a structure with a higher crystalline region after the treatment. Thus, the structure was a more compact and tight structure, which became difficult to attack [2].

## 3. Materials and Method

### 3.1. Pretreatment of Microcrystalline Cellulose (MCC)

MCC was purchased from Sigma Aldrich. Iron (III) chloride hexahydrate and hydrochloric acid fuming 37% were of analytical grade and bought from R & M Chemicals and Merck, respectively. Different concentrations of FeCl_3_ solution (0.66–2.54 M) were prepared in 2.5 M HCl acid. Fifteen milliliters of this prepared solution were taken in 50-mL round bottomed flask. One gram of MCC sample was added and stirred in a magnetic hot plate. The flasks were heated to various temperatures and reaction times in a constant temperature oil bath. Samples were withdrawn from the reaction mixture under different reaction conditions preset by the experimental design (Table 1 and Table 2). The resulting suspension was centrifuged several times at 6000 rpm and washed with distilled deionized water. A certain portion of the suspension was dried up to a constant weight at 105 °C, and the yield of the treated sample was calculated according to the following equation:

(4) $$Yield\%=\;\frac{M_{1}\;\times M_{3}}{M_{2}\times M_{0}}\;\times 100$$

Here, *M*_0_ is the initial weight of MCC taken; *M*_1_ is the mass of the dry powder finally obtained; *M*_2_ is the mass of the suspension sample used to acquire the dry powder; and *M*_3_ is the total mass of the suspension obtained in the final preparation [38]. The remaining suspension was dried by a freeze dryer, and the dried sample was stored for subsequent characterization.

### 3.2. Experimental Design

The influence of three sovereign variables, *x*_1_ (temperature), *x*_2_ (time) and *x*_3_ (concentration of FeCl_3_ catalyst), on two responses, *y*_1_ and *y*_2_, namely the percentage of crystallinity and yield, respectively, were determined. A two-level central composite design (CCD) using the response surface methodological approach (RSM) was undertaken to analyze the main and combined effects of variables on the responses studied, as well as to develop models with the subsequent optimization of the process. Therefore, 20 experimental runs were required based on the second-order CCD with three independent variables. The independent variable ranges studied were temperature (56.14–173.86 °C), time (1.98–7.02 h) and FeCl_3_ concentration (0.66–2.34 M), while HCl concentration was fixed at 2.5 M. Experimental runs were randomized to reduce the effects of inexplicable inconsistency in the actual responses due to peripheral factors. At the center point, 6 experiments were conducted at identical conditions to calculate the repeatability of the data [39,40]. The complete design matrix conforming to the levels of preferred variables is illustrated by Table 5 and Table 6. Based on Table 5 and Table 7, the prearrangement of experimental design permitted the development of the pertinent empirical equations [41].

**Table 7:** Independent variables for acid hydrolysis with their actual and coded levels.

Variables	Code	Units	Coded variable levels				
Temperature	*x*_1_	°C	−α	−1	0	+1	+α
			56.14	80	115	150	173.86
Hydrolysis time	*x*_2_	Hour	1.98	2	4.5	6	7.02
Catalyst FeCl_3_ concentration	*x*_3_	M	0.66	1	1.5	2	2.34

The independent variables are coded as the (−1, +1) interval, where the low and high levels are represented by −1 and +1, respectively. The axial points are denoted as (0, 0, ±α), (0, ±α, 0) and (±α, 0, 0). Here, α symbolizes the distance between axial points from the center (Montgomery 2001). The complete experimental matrix shown by Table 5 comprises 6 axial points, 8 factorial points and 6 center points, where the experiments were conducted in identical conditions to determine the residual error.

### 3.3. Statistical Analysis

Regression analysis was carried out for fitting the mathematical models with the experimental data and to determine the regression coefficients. The statistical significance of the model terms was obtained by the analysis of variance (ANOVA) test. The process parameters were optimized to get the inclusive ideal region where the responses under consideration would be maximal. The performance of the response surface was investigated by using the regression polynomial equation. The generalized polynomial model proposed can be expressed as:

(5) $$Y_{i\;}=\;\beta_{0\;}+\beta_{1}x_{1\;}+\;\beta_{2}x_{2}+\;\beta_{3}x_{3\;}+\beta_{11}x_{1\;}^{2\;}+\;\beta_{22}x_{2}^{2}+\beta_{33}x_{3}^{2}+\beta_{12}x_{1}x_{2}+\beta_{13}x_{1}x_{3}+\beta_{23}x_{2}x_{3}$$

where *Y_i_* is the desired response; β_0_ is the constant term; β_1_, β_2_ and β_3_ are the regression coefficients for the linear effect terms; β_11_, β_22_ and β_33_ are the quadratic effects; and β_12_, β_13_ and β_23_ are the interaction effects. Here, in this model, *x*_1_, *x*_2_ and *x*_3_ are the process variables.

The ANOVA analysis of the experimental runs provided the regression coefficients for linear, quadratic and interaction terms individually. The significance of each term for the responses was also evaluated by observing the *F*-ratio, where the probability (*p*) is less than 0.05. The adequacy of the models was determined using model analysis, a lack-of-fit test and coefficient of determination (*R*^2^) analysis. The experimental design matrix, data analysis and optimization procedure were performed using the Design of Experiment statistical package 7.

### 3.4. Optimization Process

After multiple regression analysis and the ANOVA test for the developed models related to the crystallinity index (*y*_1_) and yield (*y*_2_), the numerical optimization procedure was performed. This gave the optimal levels of three factors (*x*_1_, *x*_2_ and *x*_3_) to obtain the highest crystallinity percentage with the maximal yield. The goal for each variable was kept within the studied range. Furthermore, a graphical technique in terms of 3D response surface plots was used to visualize the rapport between the responses and experimental levels of each variable, where one variable was kept constant at the center point, whereby another two variables were varied within the experimental range.

### 3.5. Verification of Developed Models

The adequacy of the developed models for the responses was verified by conducting experiments under the optimum conditions suggested by the software. The experimental and predicted values of the responses were compared, and the percentage of error was calculated in order to check the validity of the predicted models.

### 3.6. Physiochemical Characterization of Pretreated Microcrystalline Cellulose (MCC)

A scanning electron microscope (FEI Quanta 200F) was used to obtain the surface morphological features of the MCC and the pretreated MCC under optimum conditions. The surface functional groups of the MCC and pretreated MCC were detected by a Fourier transform infrared (FTIR) spectroscope (FTIR-Bruker IFS 66/S). The spectra were recorded from 4,000 to 400 cm^−1^. The crystalline structure of the samples was analyzed by using Cu-Kα radiation sources by using XRD (Bruker AXSD8 Advance). The percentage of crystallinity was calculated according to Equation (6) [42]:

(6) $$C=\;\frac{I_{002}-\;I_{am}}{I_{002}}\times 100$$

Here, *C* is the percentage of crystallinity; *I*_002_ is the maximum intensity of the 002 peak at 2θ = 22.5° and *I_am_* is the intensity at 2θ = 18.7°.

Proximate analysis was carried out using thermal gravimetric analysis (TGA) equipment (Model Mettler Tolodo TGA/SDTA 851^e^) to determine the weight loss of the MCC and the pretreated MCC under optimum conditions.

## 4. Conclusions

Acid hydrolysis in the presence of FeCl_3_ catalyst can be used for the selective hydrolysis of MCC. The addition of FeCl_3_ can ensure the hydrolysis of amorphous regions to a greater extent. X-ray diffraction results showed that the percentage of crystallinity was increased due to acid hydrolysis treatment. The process parameters were optimized by using RSM. The factors affecting the percentage of crystallinity and yield were in the order of: temperature > time > catalyst concentration. FeCl_3_ plays a supplementary role in the acid hydrolysis of MCC. Model simulation and theoretical optimization were carried out. The theoretical values for the percentage of crystallinity and yield were close to the experimental one, resulting in small error percentages of 1.32% and 1.68%, respectively. Thus, it can be concluded that the RSM technique based on CCD design is suitable for identifying and optimizing the variables influencing the catalytic acid hydrolysis of MCC.
